# Oxidative Stress and Inflammatory Modulation of Ca^2+^ Handling in Metabolic HFpEF-Related Left Atrial Cardiomyopathy

**DOI:** 10.3390/antiox9090860

**Published:** 2020-09-14

**Authors:** David Bode, Yan Wen, Niklas Hegemann, Uwe Primessnig, Abdul Parwani, Leif-Hendrik Boldt, Burkert M. Pieske, Frank R. Heinzel, Felix Hohendanner

**Affiliations:** 1 Department of Internal Medicine and Cardiology, Campus Virchow-Klinikum, Charité University Medicine, 13353 Berlin, Germany; david.bode@charite.de (D.B.); wenyanxmu@outlook.com (Y.W.); niklas.hegemann@charite.de (N.H.); uwe.primessnig@charite.de (U.P.); abdul.parwani@charite.de (A.P.); leif-hendrik.boldt@charite.de (L.-H.B.); burkert.pieske@charite.de (B.M.P.); frank.heinzel@charite.de (F.R.H.); 2 German Center for Cardiovascular Research (DZHK), Partner Site Berlin, 10785 Berlin, Germany; 3 Berlin Institute of Health (BIH), 10178 Berlin, Germany; 4 Department of Internal Medicine and Cardiology, German Heart Center Berlin, 13353 Berlin, Germany

**Keywords:** inflammation, reactive oxygen species, atrial cardiomyopathy, heart failure with preserved ejection fraction (HFpEF), metabolic syndrome, excitation-contraction coupling, calcium, left atrial cardiomyocytes

## Abstract

Metabolic syndrome-mediated heart failure with preserved ejection fraction (HFpEF) is commonly accompanied by left atrial (LA) cardiomyopathy, significantly affecting morbidity and mortality. We evaluate the role of reactive oxygen species (ROS) and intrinsic inflammation (TNF-α, IL-10) related to dysfunctional Ca^2+^ homeostasis of LA cardiomyocytes in a rat model of metabolic HFpEF. ZFS-1 obese rats showed features of HFpEF and atrial cardiomyopathy in vivo: increased left ventricular (LV) mass, E/e’ and LA size and preserved LV ejection fraction. In vitro, LA cardiomyocytes exhibited more mitochondrial-fission (MitoTracker) and ROS-production (H2DCF). In wildtype (WT), pro-inflammatory TNF-α impaired cellular Ca^2+^ homeostasis, while anti-inflammatory IL-10 had no notable effect (confocal microscopy; Fluo-4). In HFpEF, TNF-α had no effect on Ca^2+^ homeostasis associated with decreased TNF-α receptor expression (western blot). In addition, IL-10 substantially improved Ca^2+^ release and reuptake, while IL-10 receptor-1 expression was unaltered. Oxidative stress in metabolic syndrome mediated LA cardiomyopathy was increased and anti-inflammatory treatment positively affected dysfunctional Ca^2+^ homeostasis. Our data indicates, that patients with HFpEF-related LA dysfunction might profit from IL-10 targeted therapy, which should be further explored in preclinical trials.

## 1. Introduction

In heart failure (HF) with preserved ejection fraction (HFpEF), patients suffer from typical HF symptoms (i.e., dyspnea, reduced exercise capacity), while presenting normal left ventricular ejection fraction (>50%). HFpEF is increasingly prevalent and currently accounts for approximately half of all HF patients [1]. While pharmacologic treatment options for HF with reduced ejection fraction (HFrEF) evolved notably during last decades, drug therapy for HFpEF remains elusive, despite high morbidity and mortality [2,3]. One reason for failure of large clinical trials in HFpEF may be attributed to the complex and heterogenous etiology of the disease. A broad spectrum of systemic triggers have been have been identified in HFpEF, such as a pro-inflammatory state, arterial and microvascular dysfunction, fluid retention, impaired systolic and diastolic function, cardiac hypertrophy, energetic abnormalities and interstitial cardiac fibrosis [4].

The prevalence of co-morbidities which are marked by endothelial dysfunction and systemic inflammation, such as obesity, diabetes mellitus type 2, arterial hypertension, dyslipidemia and renal dysfunction, is strikingly high amongst HFpEF patients [5]. Mechanical stress and systemic inflammation have been shown stimulate the production of reactive oxygen species (ROS) of cardiac endothelial cells in HFpEF with detrimental effects on adverse myocardial remodeling and cardiac contractility [6,7,8]. 

Antioxidative therapies to treat cardiovascular disease have been studied extensively in preclinical trials, where they have shown promising results. Treatment regimen have either been focused on inhibiting ROS production, improving endogenous antioxidant capacity, or supplementing exogenous antioxidants [9]. Yet, early large-scale clinical trials have disappointed. The question remains, whether an alternative approach targeting oxidative stress would prove more effective in a clinical setting: Inflammation and oxidative stress are mutually interdependent [10] and increased plasma cytokines (tumor necrosis factor α (TNF-α), interleukin-6) are independent predictors of mortality in advanced HF [11]. The Cannakinumab Anti-Inflammatory Thrombosis Outcomes Study (CANTOS) trial has served the first proof of an effective anti-inflammatory therapy (anti-interleukin-1β) in HF [12,13]. HFpEF is commonly accompanied by left atrial cardiomyopathy, an independent predictor of mortality [14]. HFpEF-related left atrial cardiomyopathy is characterized by structural (e.g., enlargement, fibrosis) and functional remodeling (i.e., contractile dysfunction, arrhythmic disorders, endocrine dysregulation) [15]. Enlargement of left atria predicts the new-onset of atrial fibrillation and HF [16]. Impairment of left atrial contractile function is an independent predictor of mortality in HF [17]. The mechanisms underlying HFpEF-related left atrial cardiomyopathy remain poorly understood. However, especially oxidative stress has also been shown to be pivotal for left atrial electrical (i.e., atrial fibrillation) and mechanical (i.e., impaired contractile function) remodeling [18]. We have previously identified neurohumoral activation (angiotensin-2) and paracrine activation through the fibroblast secretome as pivotal disruptors of Ca^2+^ handling in metabolic HFpEF-related left atrial cardiomyopathy [19,20]. Pharmacological approaches targeting oxidative stress and inflammation for the treatment of heart failure and associated atrial cardiomyopathy remain elusive.

In this study, we investigate the role of myocardial inflammation through TNF-α/interleukin-10 (IL-10) signaling for dysfunctional Ca^2+^ cycling of cardiomyocytes in a rat model of metabolic HFpEF-related left atrial cardiomyopathy. In addition, we explore the production of ROS as a result of disrupted mitochondrial energy metabolism.

## 2. Methods

### 2.1. Heart Failure (HF) Model

Experiments involving animals were approved by German authorities (G0276/16, G0317/17). The ZSF-1 obese rat model is based on a leptin receptor mutation resulting in a metabolic syndrome-related HFpEF phenotype [21] associated with atrial remodeling as reported previously [20]. Wild-type (WT) rats (Wistar Kyoto and HFpEF (ZSF-1 obese) animals were obtained at 10 weeks (Charles River Laboratories, Wilmongton, NC, USA) and fed a high caloric diet (Purina 5008; LabDiet, St. Louis, MO, USA). Final experiments were performed between 23 (Figure 2) and 27 weeks (remainder).

### 2.2. Echocardiography

Echocardiography was performed and analyzed as previously described [20] by an experienced observer (N.H.) using a Vevo lab ultrasound system (Visual Sonics, Toronto, ON, Canada).

### 2.3. Chemicals, Solutions

All chemicals were acquired from Sigma-Aldrich (St. Louis, MO, USA) unless declared otherwise. Fluorescent dyes Fluo-4 AM, MitoTracker red and 2’,7’-Dichlordihydrofluorescein-diacetat (H2-DCF) were acquired from Thermo Fisher Scientific (Waltham, MA, USA). Tyrode solution was composed of: 130 mM NaCl, 4 mM KCl, 2 mM CaCl_2_, 1 mM MgCl_2_, 10 mM Glucose and 10 mM HEPES; the pH was adjusted to 7.4 at 37 °C with NaOH. Glass coverslips were coated with laminin and allowed to dry prior to cell plating. Fluorescent dyes were used at the following concentration (in µM): 5 Fluo-4, 1 Mitotracker red, 10 H2-DCF.

### 2.4. Cardiomyocyte Isolation

Primary left atrial cardiomyocytes were isolated as described previously [22]. In brief, the rat heart was excised and mounted to a Langendorff-apparatus (self assembly). Pressure of the left atria was manipulated using a novel approach as reported previously [22]. Myocardial tissue was digested using Ca^2+^ free solution containing highly purified collagenase. Isolated left atrial cardiomyocytes were readjusted to Ca^2+^ in a stepwise manner and placed on laminin-coated dishes for sub-sequent experiments.

### 2.5. Mitochondrial Structure

Left atrial cardiomyocytes were loaded with Mitotracker red, transferred to an LSM 800 laser scanning microscope (Carl Zeiss AG, Oberkochen, Germany) and two-dimensional images acquired (excitation 561 nm, emission: 565–700 nm). Binary images representing the presence and absence of mitochondria were derived using a 2-step Otsu thresholding algorithm [23]. The perimeter of mitochondrial structures was quantified by edge detection. For each cell, the ratio of the total mitochondrial perimeter and the total mitochondrial surface area was calculated as an indicator of mitochondrial fission. 

### 2.6. Reactive Oxygen Species (ROS)

Left atrial cardiomyocytes were loaded with H2-DCF and transferred to an LSM 800 laser scanning microscope. A time series of 15 two-dimensional images were recorded (excitation 488 nm, emission: 505–525 nm; interval 2 s). Signal intensity of the initial image was defined as *F*_0_, production of ROS calculated as Δ*F* = *F* − *F*_0_ per image, averaged per image sequence and reported as the respective rate Δ*F*/(*F*_0_ × *t*).

### 2.7. Ca^2+^ Imaging

Left atrial cardiomyocytes were incubated with Tyrode solution in the presence or absence of TNF-α (10 nM) or IL-10 (10 nM) at 37 °C for 30 min. Sub-sequently, cells were loaded with Fluo-4 for another 30 min at 37 °C (in the continuous presence of the respective cytokine). The cells were then washed twice with Tyrode solution and transferred to an LSM 800 laser scanning microscope. Experiments were conducted at 37 °C at steady state following approximately 1 min of electric stimulation at 1 Hz. Ca^2+^ transients (CaT) were recorded for 10 s and averaged prior to analysis. Time-dependent changes of cytosolic [Ca^2+^] during electric pacing are reported as Δ*F/F*_0_. Tau of decay (mono-exponential fit) is reported as a mesuare of Ca^2+^ reuptake. For early Ca^2+^ release (ER) site analysis: F50 was defined as 50% of the CaT amplitude and the corresponding time-to-F50 (TF50) calculated as an indicator of ER. Scan lines along the longitudinal axis were grouped into 1 µm intervals, indicating active couplons [24,25]. ER was defined to be smaller than the average TF50 of the control group (<15.0 ms) and an ER site was defined to be an active couplon with ER events in at least two out of six consecutive stimulation cycles. The fraction of ER events ER sites in six consecutive cycles was quantified as the probability of ER.

### 2.8. Western Blots

Left atrial tissue was homogenized in lysis buffer (composition (in mM): 20 Tris-HCl, 137 NaCl, 20 NaF, 1 sodium orthovandate, 1 sodium pyrophosphate, 50 β-glycerophosphate, 10 EDTA, 1 EGTA, 1 PMSF, 10% glycerol, 1% NP 40, 4 µg/mL aprotinin 4 µg/mL pepstatin A, 4 µg/mL leupeptin. Lysed tissue was run on 16.5% Tricine gels and transferred to a nitrocellulose membrance for 120 min. Total protein was stained with Ponceau S. Blocking of non-specific binding was performed with 5% dried milk in Tris-buffered salin with 0.1% Tween-20. Membranes were probed with anti-TNF-α receptor 1/2, anti-IL-10 receptor 1/2 (Santa Cruz Biothecnology, Santa Cruz, CA, USA) and anti-GAPDH (Abcam, Cambridge, UK) for 12 h at 4 °C. Secondary antibodies were either anti-mouse IgG (680RD, IRDye; LI-COR Biosciences, Lincoln, NE, USA) or anti-rabbit IgG (800CW, LI-COR Biosciences, Lincoln, NE, USA). The signal was recorded with an Odyssey CLx System (LI-COR Biosciences, Lincoln, NE, USA) and quantified with the LI-COR Image Studio software (LI-COR Biosciences, Lincoln, NE, USA).

### 2.9. Statistical Analysis

Results are shown as mean ± standard error. Statistical tests and *p*-values are reported in the figure legend. A *p*-value below 0.05 was assumed to be of statistical significance.

## 3. Results

### 3.1. ZSF-1 Obese Rats Show Distinct Features of Metabolic HFpEF-Related Left Atrial Cardiomyopathy

**Figure 1:**
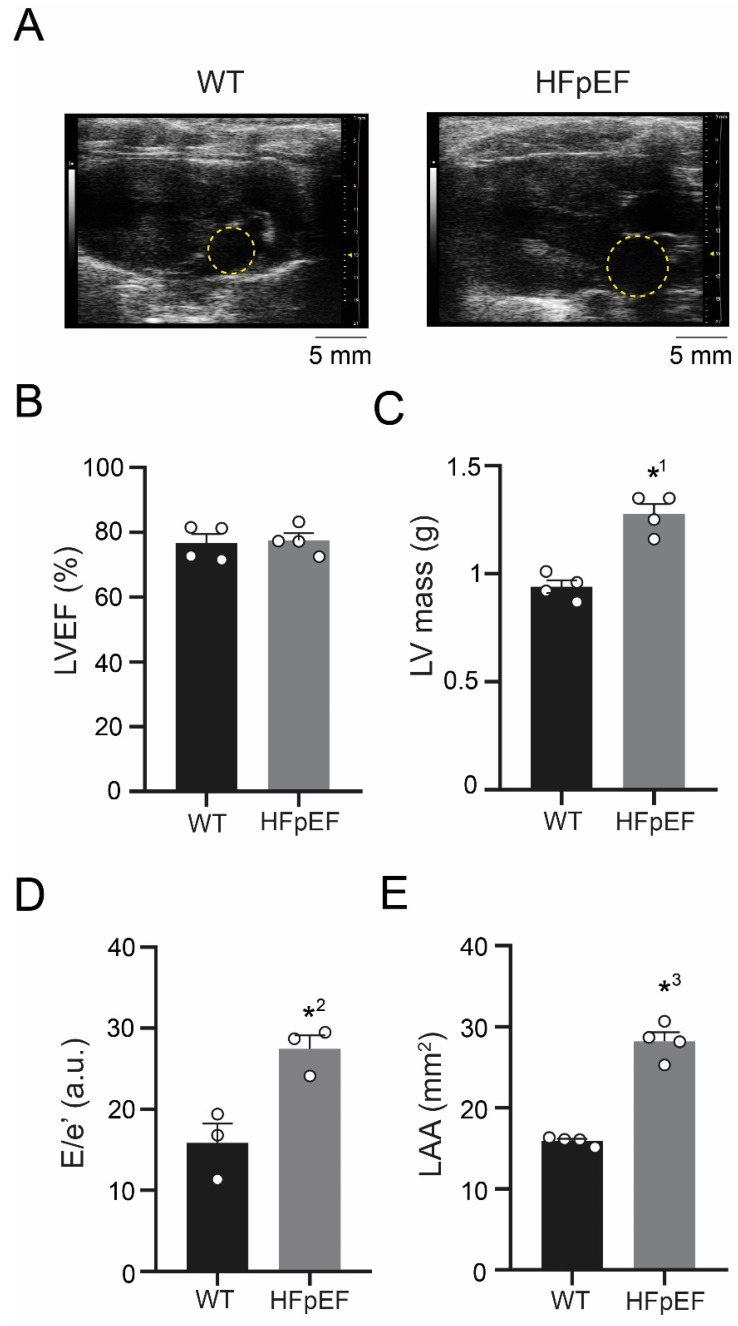
(**A**) Representative example of transthoracic echocardiographic assessment (B-mode) in parasternal long axis view of WT (left) and HFpEF (right) during diastole, yellow circles indicate left atria. (**B**) Corresponding left ventricular ejection fraction and (**C**) left ventricular mass (weighed). (**D**) E/e’ as a measure of diastolic dysfunction (derived from mitral valve flow and tissue Doppler). (**E**) Left atrial area. Statistical analysis: Student’s *t*-test. *p*-values: *^1^ 0.0008, *^2^ 0.016, *^3^ <0.0001. *n*-numbers indicate animals.

Left ventricular function was assessed in vivo with echocardiography (Figure 1A) and left ventricular mass weighed post-mortem. Left ventricular ejection fraction remained preserved (Figure 1B), while left ventricular mass was increased (Figure 1C). Combined mitral valve and tissue Doppler analysis yielded an increased E/e’ ratio in HFpEF, a measure of diastolic dysfunction (Figure 1D). Left atria were severely enlarged in HFpEF, indicating advanced adverse remodeling (Figure 1E).

### 3.2. HFpEF Shows Increased Mitochondrial Fission and Production of ROS

**Figure 2:**
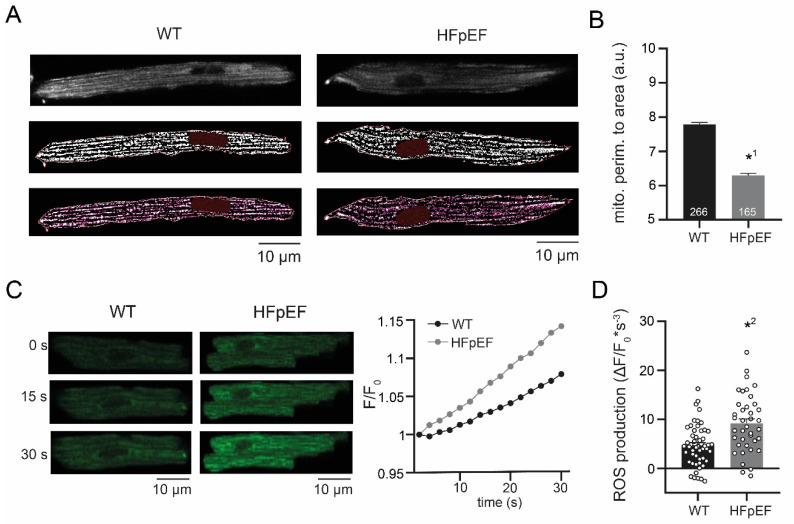
(**A**) Representative example of mitochondrial structure (dye: Mitotracker) in left atrial cardiomyocytes (upper), after thresholding (center) and after contour analysis (lower). (**B**) Corresponding data of structural perimeter to area ratio as an indicator mitochondrial fission. (**C**) Representative example of ROS measurements (left; dye: H2DCF) and averaged trace (all cells per group). (**D**) Corresponding data of ROS production. Statistical analysis: Student’s *t*-test. *p*-values: *^1^ <0.0001, *^2^ <0.0001. *n*-numbers indicate cells (from four animals per group).

To investigate the contribution of mitochondrial function to the pathology of HFpEF-related atrial cardiomyopathy, left atrial cardiomyocytes were isolated and mitochondria examined in vitro. Structural analysis of mitochondria revealed a reduced perimeter to area ratio in HFpEF, indicating enhanced mitochondrial fission (Figure 2A,B). The rate of oxidation was measured to evaluate mitochondrial function. In accordance with increased mitochondrial fission, left atrial cardiomyocytes in HFpEF also showed an increased production of ROS (Figure 2C,D).

### 3.3. Ca^2+^ Signaling during Excitation-Contraction Coupling in HFpEF Is Not Affected by TNF-α, but Benefits from Exposure to IL-10

**Figure 3:**
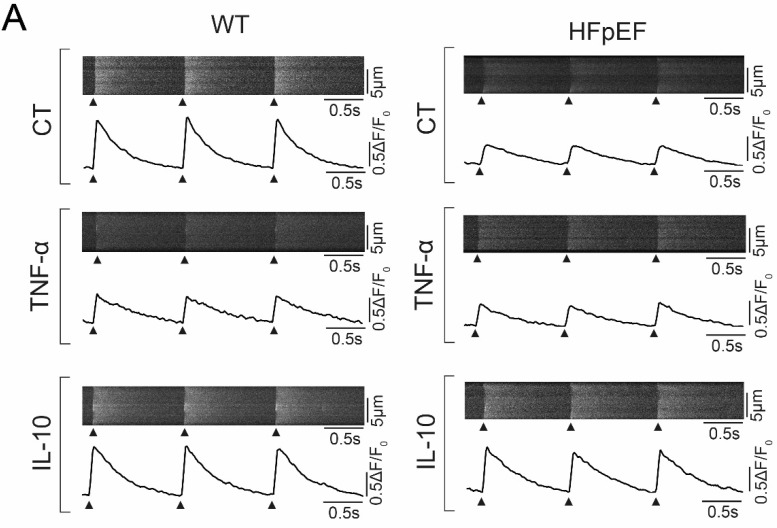

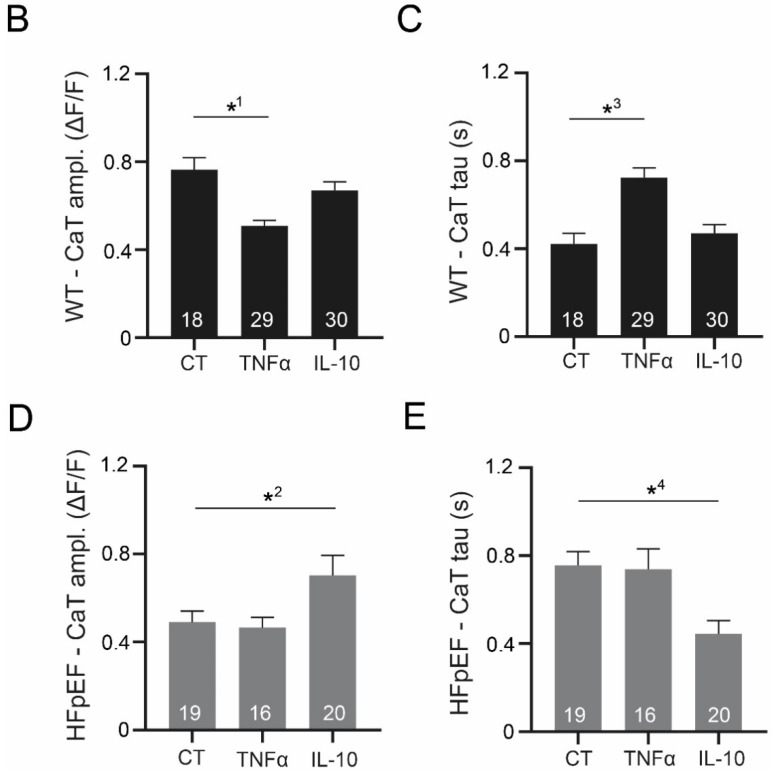
(**A**) Representative example of three consecutive CaT of left atrial cardiomyocytes after 1 h incubation in Tyrode solution (CT; upper), with additional TNF-α (center) or IL-10 (lower; 10 μM, respectively) in WT (left) and HFpEF (right) at 1 Hz electric stimulation (black arrows indicate stimulation triggers). (**B**) Corresponding CaT amplitude and (**C**) tau of decay of WT. (**D**) Corresponding CaT amplitude and (**E**) tau of decay of HFpEF. Statistical analysis: One-way ANOVA, post-hoc Bonferroni. *p*-values: *^1^ <0.0001, *^2^ <0.0001, *^3^ 0.0175, *^4^ 0.0009. *n*-numbers indicate cells (from four animals per group).

In order to assess inflammatory modulation of Ca^2+^ cycling in HFpEF, CaT of left atrial cardiomyocytes were recorded after exposure to TNF-α and its anti-inflammatory counterpart IL-10 (Figure 3A). In WT, TNF-α led to a significant reduction of CaT amplitudes (Figure 3B), which was linked to a deceleration of Ca^2+^ removal (Figure 3C). Anti-inflammatory treatment with IL-10 did not show an effect on CaT in WT (Figure 3B,C). Interestingly, a diametral effect of IL-10 could be observed in HFpEF. IL-10 mediated an increased CaT amplitude (Figure 3D), linked to an accelerated Ca^2+^ removal (Figure 3E), while TNF-*α* did not have an effect on CaT (Figure 3D,E).

### 3.4. IL-10 Induces Recruitment and Synchronization of Early Ca^2+^ Release Sites in HFpEF

**Figure 4:**
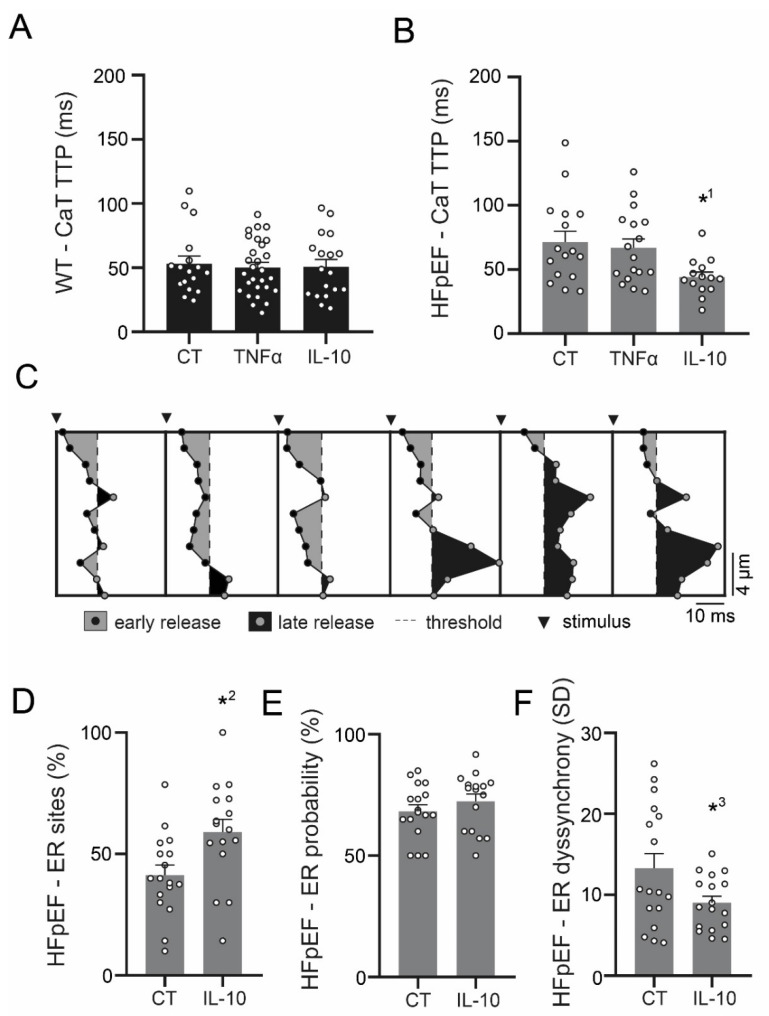
(**A**) CaT time-to-peak (TTP) of left atrial cardiomyocytes in WT and (**B**) HFpEF after 1 h incubation in Tyrode solution (CT), with additional TNF α or IL-10 (10 μM, respectively). (**C**) Example visualization of early Ca^2+^ release (ER) site analysis. (**D**) Corresponding data of fractional ER sites (E; > 3 ER events in 10 consecutive cycles). (**E**) Respective probability of ER of ER sites. (**F**) Standard deviation (SD) of ER sites as a measure of synchrony. Statistical analysis: One-way ANOVA, post-hoc Bonferroni (**A**,**B**) or student’s *t*-test (**D**–**F**). *p*-values: *^1^ 0.015, *^2^ 0.012, *^3^ 0.039. *n*-numbers indicate cells (from four animals per group).

Neither TNF-α, nor IL-10 had an effect on Ca^2+^ release in WT (Figure 4A). In HFpEF however, IL-10 significantly accelerated Ca^2+^ release (Figure 4B). An in-depth analysis of early Ca^2+^ release (ER) sites was conducted to evaluate the origin of improved Ca^2+^ release (Figure 4C). The positive effect was found to be mediated by an improved recruitment of ER sites (Figure 4D), rather than an increased overall probability of ER (Figure 4E). In addition, IL-10 synchronized ER sites (Figure 4F).

### 3.5. TNF-α Receptor Expression Is Reduced, IL-10 Receptor Expression Partially Maintained in HFpEF

**Figure 5:**
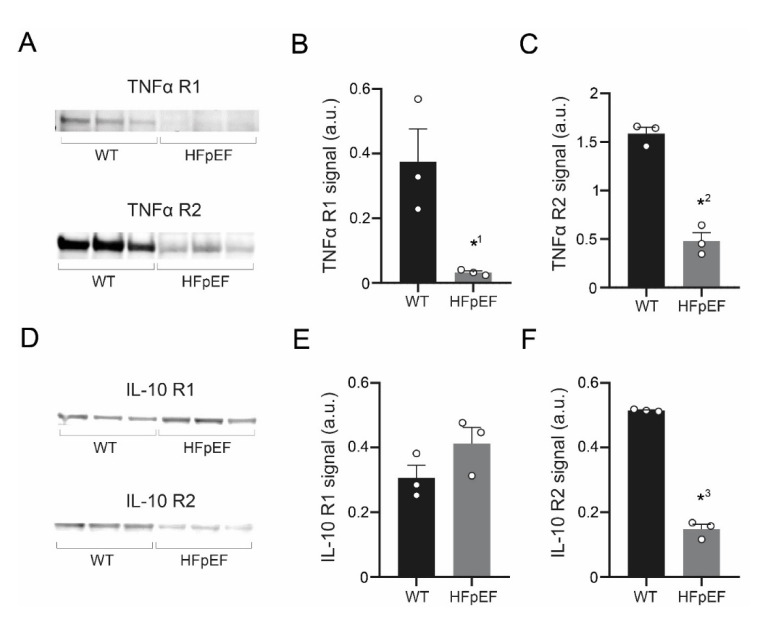
(**A**) Western Blot of TNF-α receptor type 1 (TNF-α R1; upper) and TNF-α receptor type 2 (TNF-α R2; lower) in left atrial myocardial tissue. (**B**) Corresponding data of TNF-α R1 and (**C**) TNF-α R2. (**D**) Western Blot of IL-10 receptor type 1 (IL-10 R1; upper) and IL-10 receptor type 2 (IL-10 R2; lower). (**E**) Corresponding data of IL-10 R1 and (**F**) IL-10 R2. Statistical analysis: Student’s *t*-test. *p*-values: *^1^ 0.027, *^2^ 0.0005, *^3^ <0.0001. *n*-numbers indicate animals.

To further explore the contrasting effects of TNF-α and IL-10 on Ca^2+^ signaling during excitation-contraction coupling in HFpEF, protein expression of their respective receptors was quantified by Western Blot analysis. Left atrial tissue in HFpEF showed a significant down regulation of TNF-α receptors type 1 and type 2 (Figure 5A–C). IL-10 receptor expression was partially maintained in HFpEF: type 1 expression was unchanged, while type 2 was significantly lower (Figure 5D–F).

## 4. Discussion

Left atrial cardiomyocytes exhibited an increased production of ROS in a rat model of metabolic HFpEF-related atrial cardiomyopathy, which was associated with enhanced mitochondrial fission. TNF-α severely impaired Ca^2+^ uptake and CaT release amplitudes only in WT, yet it had no effect on left atrial cardiomyocytes in HFpEF. However, CaT amplitudes and Ca^2+^ uptake were improved by anti-inflammatory treatment with IL-10. In addition, IL-10 enhanced recruitment of ER sites in HFpEF, thus accelerating and synchronizing Ca^2+^ release. Left atrial cardiomyocytes in HFpEF showed reduced expression of TNF-α receptors, while expression of IL-10 receptor type 1 remained intact.

Abnormal Ca^2+^/Na^+^ handling and oxidative stress are important, closely intertwined pathophysiological hallmarks of HF [26,27]. Dysfunctional excitation-contraction coupling demands an increased energy production, which leads to oxidative stress once the buffering capacity of the antioxidant defense system has been exceeded. In addition, an altered cellular Ca^2+^/Na^+^ homeostasis may directly affect mitochondrial [Ca^2+^] uptake and regulate mitochondrial metabolism [28]. In turn, ROS can trigger Ca^2+^-mediated cellular arrhythmias, i.e., via abnormal Ca^2+^ release from the sarcoplasmic reticulum (resulting in delayed after depolarizations [29]), or via stimulation of l-type Ca^2+^ current (early after depolarizations) [30]. In this context, the discovery of an increased ROS production of left atrial cardiomyocytes in the setting of metabolic syndrome-related HFpEF (Figure 2) is of particular importance. The prevalence of atrial fibrillation in HFpEF has been reported as high as 39% (12% higher than HFrEF) [31] and ROS may serve as a promising target to reduce the burden of atrial electrical dysfunction (i.e., arrhythmias) in HFpEF.

**Figure 6:**
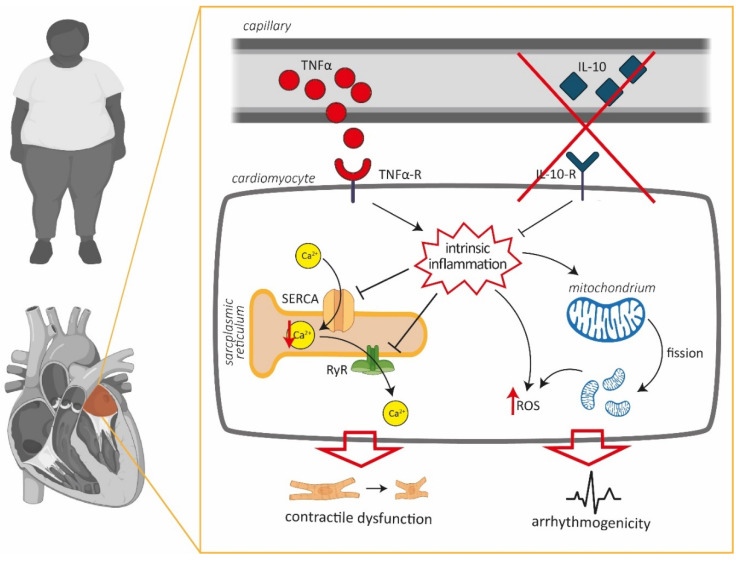
Proposed effect of dysfunctional TNFα/IL-10 signaling in cardiomyocytes during metabolic HFpEF-related left atrial cardiomyopathy. Intrinsic inflammation mediates impairment of cellular Ca^2+^ homeostasis and facilitates enhanced production of reactive oxygen species (ROS).

Our experimental data indicates that Ca^2+^ handing of left atrial cardiomyocytes in HFpEF may be impaired associated with an upregulation of inflammatory pathways (Figure 6). In WT, left atrial cardiomyocytes responded to TNF-α with decelerated Ca^2+^ removal and a decreased CaT amplitude (Figure 3), in accordance with previous reports of TNF-α induced downregulation of sarcoplasmic/endoplasmic reticulum calcium ATPase (SERCA) 2a [32]. In HFpEF however, TNF-α did not affect Ca^2+^ handling. However, left atrial cardiomyocytes responded with an accelerated Ca^2+^ uptake and an increased CaT amplitude in response to IL-10. IL-10 directly attenuates TNF-α induced nuclear factor ‘kappa-light-chain-enhancer’ of activated B-cells (NFκB) pathway activation by upregulating extracellular signal-regulated kinase 1/2 (ERK 1/2) mitogen-activated protein kinase (MAPK) phosphorylation [33], which has also been reported as effectively countering TNF-α induced oxidative stress in cardiomyocytes [34]. In addition to improving Ca^2+^ removal and CaT amplitudes, IL-10 also facilitated a notable acceleration and synchronization of Ca^2+^ release kinetics (Figure 4).

From these findings, two plausible approaches to anti-inflammatory treatment of metabolic HFpEF-related atrial cardiomyopathies can be derived: antagonizing TNF-α (i.e., lowering circulating cytokines, receptor blockage at effector site) or agonizing IL-10 (i.e., exogenous substitution). Large clinicals trials evaluating TNF-α inhibitors in HF (Randomised Etanercept Worldwide Evaluation (RENEWAL) [35], Anti-TNF Therapy Against Congestive Heart Failure (ATTACH) [36]) have yielded dissatisfactory results and further testing of TNF-α inhibitors in patients seems currently unlikely. The lacking efficacy of TNF-α inhibitors can be partly supported by the experimental data of this study: at the time of disease manifestation, cardiomyocytes have already entered an ‘inflamed state’ through NFκB activation and have reduced TNF-α receptor expression (Figure 5), thus removing the beneficial effector site of lower TNF-α levels. However, anti-inflammatory therapy with IL-10 for the treatment of HF seems plausible and has not previously been tested in clinical trials. Expression of IL-10 receptor type 1 was maintained and a satisfactory attenuation of Ca^2+^ handling could be observed upon IL-10 treatment. Previous studies with patients suffering from Crohn’s disease have reported subcutaneous therapy with recombinant IL-10 to be safe in humans [37]. However, further animal studies (i.e., isolated hearts, gene knockouts) need to be performed in order to confirm the present findings before translational in vivo studies can be performed.

## 5. Conclusions

Cardiomyocytes in metabolic HFpEF-related atrial cardiomyopathy exhibit mitochondrial fission, increased oxidative stress and dysfunctional Ca^2+^ handling. Anti-inflammatory treatment with IL-10 facilitates an attenuation of Ca^2+^ signaling of left atrial cardiomyocytes in HFpEF. IL-10 substitution might pose a viable treatment strategy for atrial dysfunction (arrhythmias, contractile dysfunction) in the setting of metabolic syndrome-related HFpEF and its efficacy should be further evaluated in preclinical trials.
